# Effects of low temperature on photoinhibition and singlet oxygen production in four natural accessions of *Arabidopsis*

**DOI:** 10.1007/s00425-020-03423-0

**Published:** 2020-07-15

**Authors:** Heta Mattila, Kumud B. Mishra, Iiris Kuusisto, Anamika Mishra, Kateřina Novotná, David Šebela, Esa Tyystjärvi

**Affiliations:** 1 Department of Biochemistry, Molecular Plant Biology, University of Turku, 20014 Turku, Finland; 2 Global Change Research Institute of the Czech Academy of Sciences, Bělidla 986, 4a, Brno, 603 00 Czech Republic

**Keywords:** Acclimation, Charge recombination, Chilling stress, Cold-hardening, Photodamage, Photoinactivation, Reactive oxygen species, SOSG

## Abstract

**Main conclusions:**

Low temperature decreases PSII damage in vivo, confirming earlier in vitro results. Susceptibility to photoinhibition differs among Arabidopsis accessions and moderately decreases after 2-week cold-treatment. Flavonols may alleviate photoinhibition.

**Abstract:**

The rate of light-induced inactivation of photosystem II (PSII) at 22 and 4 °C was measured from natural accessions of *Arabidopsis thaliana* (Rschew, Tenela, Columbia-0, Coimbra) grown under optimal conditions (21 °C), and at 4 °C from plants shifted to 4 °C for 2 weeks. Measurements were done in the absence and presence of lincomycin (to block repair). PSII activity was assayed with the chlorophyll *a* fluorescence parameter *F*_v_/*F*_m_ and with light-saturated rate of oxygen evolution using a quinone acceptor. When grown at 21 °C, Rschew was the most tolerant to photoinhibition and Coimbra the least. Damage to PSII, judged from fitting the decrease in oxygen evolution or *F*_v_/*F*_m_ to a first-order equation, proceeded more slowly or equally at 4 than at 22 °C. The 2-week cold-treatment decreased photoinhibition at 4 °C consistently in Columbia-0 and Coimbra, whereas in Rschew and Tenela the results depended on the method used to assay photoinhibition. The rate of singlet oxygen production by isolated thylakoid membranes, measured with histidine, stayed the same or slightly decreased with decreasing temperature. On the other hand, measurements of singlet oxygen from leaves with Singlet Oxygen Sensor Green suggest that in vivo more singlet oxygen is produced at 4 °C. Under high light, the PSII electron acceptor *Q*_A_ was more reduced at 4 than at 22 °C. Singlet oxygen production, in vitro or in vivo, did not decrease due to the cold-treatment. Epidermal flavonols increased during the cold-treatment and, in Columbia-0 and Coimbra, the amount correlated with photoinhibition tolerance.

**Electronic supplementary material:**

The online version of this article (10.1007/s00425-020-03423-0) contains supplementary material, which is available to authorized users.

## Introduction

Photosystem II (PSII) is constantly damaged by light, and synthesis of a new D1-protein is needed for the recovery of the electron transfer activity (for reviews, see Tyystjärvi 2013; Nath et al. 2013). The initial rate of the damage is directly proportional to the intensity of the illumination (Tyystjärvi and Aro 1996); if damage occurs faster than the repair, e.g. under high light, non-functional PSII units accumulate. In the literature, the term “photoinhibition” has been used to describe several phenomena; here, we strictly refer to the light-induced irreversible loss of activity of PSII. The molecular mechanism of the damage to PSII is still under debate (Tyystjärvi 2013).

Low temperature is a major factor limiting growth and geographical distribution of plant species. The repair of PSII slows down if temperature drops (Greer et al. 1986; Grennan and Ort 2007). Results about the effect of temperature on the rate of the damage itself, however, vary. A decrease in the rate of damage with a decrease in temperature in pumpkin thylakoids (Tyystjärvi et al. 1994; Tyystjärvi 2013) and chloramphenicol-treated leaves (Tyystjärvi 1993), lack of a clear temperature dependence in the cyanobacterium *Synechocystis* (Allakhverdiev and Murata 2004) and an increase in the rate of damage with decreasing temperature in the leaves of *Chenopodium album* (Tsonev and Hikosaka 2003) and *Gossypium hirsutum* (Kornyeyev et al. 2003) have been reported. Light absorption and subsequent charge separation in PSII are almost temperature-independent but, upon a sudden decrease in temperature, sink capacity decreases because carbon fixation slows down, leading to increased “excitation pressure” in the chloroplasts (Dietz et al. 1985). High excitation pressure is thought to cause photo-oxidation events leading to variegated leaf phenotypes in several mutants like *immutans* (Rosso et al. 2009). The “over-reduction” of electron transfer chain may increase singlet oxygen (^1^O_2_) production because reduced electron acceptors promote PSII charge recombination reactions. ^1^O_2_, in turn, could damage PSII (see e.g. Vass and Cser 2009). Therefore, high excitation pressure has been proposed to increase photoinhibition (Sonoike et al. 1999; Kornyeyev et al. 2003). However, these considerations do not directly probe the relationship between excitation pressure and photoinhibition of PSII.

Growth at a low temperature triggers cold-acclimation in several plant species (for a review, see Theocharis et al. 2012), causing large changes in gene expression and modifying multiple physiological processes, including synthesis of protective substances, changes in the composition of membrane lipids, and changes in enzyme activities or amounts of other substances that protect against reactive oxygen species (ROS). Light plays an important role in the development of full cold-acclimation (Soitamo et al. 2008), and it has been suggested that reduction of the photosynthetic electron transfer chain partly causes the cold-induced responses (Maxwell et al. 1995; Gray et al. 1996; Ivanov et al. 2006). Indeed, increased excitation pressure has been shown to trigger cold-acclimation responses, such as redox potential changes in PSII (see below; Sane et al. 2003).

Growth at (or long exposure to) cold (Gray et al. 1996; Krause et al. 1999; Savitch et al. 2000; Venema et al. 2000; Sane et al. 2003) or over-expression of cold-inducible genes (Yang et al. 2010) has been reported to attenuate photoinhibition of PSII at low temperatures. In some species, this is due to increased activity of the repair cycle of PSII (Krause et al. 1999; Venema et al. 2000; Grennan and Ort 2007; Rogalski et al. 2008) but also the rate of the damaging reaction has been reported to diminish due to cold-acclimation (Vonshak and Novoplansky 2008). The protection has been hypothesized to be based on the ability of cold-acclimated plants to keep the *Q*_A_ electron acceptor of PSII more oxidized in the light even at low temperatures (Öquist et al. 1993; Gray et al. 1996), thereby decreasing excitation pressure. Cold-acclimation can increase activities of the enzymes of the Calvin-Benson cycle, which increases the rate of carbon fixation at low temperatures (Strand et al. 1999). Alternative electron transfer routes (cyclic electron transfer and electron transfer to the plastid terminal oxidase) may also function more efficiently after cold-acclimation (e.g. Ivanov et al. 2012; Mishra et al. 2019). In many plant species, cold-acclimation also leads to changes in the redox potentials of the electron transport chain of PSII, possibly modifying recombination reactions and affecting ^1^O_2_ yield (Janda et al. 2000; Ivanov et al. 2001; Sane et al. 2003).

The amounts of xanthophyll pigments and/or non-photochemical quenching of excitation energy (NPQ) can increase during cold-acclimation (e.g. Krause et al. 1999; Venema et al. 2000). Furthermore, cold-acclimation may affect concentrations of anthocyanins and flavonols. Although mainly in the vacuole, flavonols have been found in chloroplasts of several species (Saunders and McClure 1976)*.* Flavonols are preferentially located at the lipid-water interphase (Scheidt et al. 2004), which allows them to quench ^1^O_2_ produced within membranes. These properties may make flavonols important scavengers of ^1^O_2_, as the lifetime of ^1^O_2_ in plant cells is so short (for reviews, see Mattila et al. 2015; Arellano and Naqvi 2016) that the damage caused by ^1^O_2_ is expected to occur near the site of origin of this ROS. In *Phillyrea latifolia* chloroplast-envelope-located flavonols were reported to quench ^1^O_2_ (Agati et al. 2007).

The capacity of plants to cold-acclimate varies greatly, but cold-tolerance is correlated with the distance of a species or accession from the equator (e.g. Hannah et al. 2006; Mishra et al. 2011). *Arabidopsis thaliana* grows over a broad geographic range with varying temperatures, and therefore, effects of low temperature and cold-acclimation on photoinhibition can be investigated in natural accessions of this model species. In the present study, we used four accessions with different freezing tolerances; the LT_50_ (lethal temperatures at which 50% of tissue damage occurs as measured by electrolyte leakage) has been reported to be − 5.7 °C, − 7.7 °C, − 5.2 °C and − 4.6 °C, for Rschew, Tenela, Columbia-0 and Coimbra, respectively (Hannah et al. 2006; Mishra et al. 2011). A shift to a cold growth temperature causes an increase in the freezing tolerance; a 2-week cold-treatment at 4 °C, as used in the present study, lowered the LT_50_ temperatures by 4.5–5.2 °C in Rschew, Columbia-0 and Tenela but only by ~ 1.5 °C in Coimbra (Hannah et al. 2006; Mishra et al. 2011). In addition, stable changes in gene expression and metabolites are observed after 2 weeks at 4 °C (Hannah et al. 2006)**.**

Previous investigations on photoinhibition of PSII at low temperatures have mostly been conducted by illuminating plants in the absence of a translation inhibitor (Gray et al. 1996; Krause et al. 1999; Sonoike et al. 1999; Venema et al. 2000; Sane et al. 2003). Thus, it is not clear whether the observed temperature dependence of photoinhibition depends on differences in the rate of damage or repair. Moreover, the effect of cold-acclimation on photoinhibition of PSII has been mostly studied only by chlorophyll *a* fluorescence (e.g. Gray et al. 1996; Krause et al. 1999; Venema et al. 2000; Sane et al. 2003). In the present study, to differentiate between NPQ, repair and damage, we illuminated leaves in the presence and absence of the chloroplast translation inhibitor, lincomycin, and assayed photoinhibition by chlorophyll *a* fluorescence as well as by oxygen evolution. In addition, ^1^O_2_ production was measured to understand the effects of cold-treatment and this ROS on photoinhibition.

## Materials and methods

### Plant material and growth conditions

*Arabidopsis thaliana* accessions, Rschew (from Russia), Tenela (Finland), Columbia-0 (Central Europe) and Coimbra (Portugal), obtained as gifts from Prof. Arnd G. Heyer, were grown in a growth chamber (FytoScope FS-RI 1600, Photon Systems Instruments, Brno, Czech Republic) for 6 weeks at day/night temperatures of 21 °C/18 °C (photosynthetic photon flux density, PPFD, of 100 µmol m^−2^ s^−1^) with ~ 60% humidity (Mishra et al. 2014). After 6 weeks of growth, half of the plants were shifted for cold-treatment to 4 °C for 2 weeks (CT) while the rest of the plants (NT) were kept at 21 °C/18 °C. In addition, Columbia-0 was shifted to 4 °C for 15 weeks after the emergency of first real leaves (CD). For growth at 4 °C and excitation pressure measurements, Columbia-0 was grown in growth chambers equipped with Osram Powerstar HQI-BT lamps at 22 °C or with fluorescent tubes at 4 °C, otherwise as previously described. To test the effect of growth light, plants (Columbia-0) were shifted under PPFD of 1000 µmol m^−2^ s^−1^ (Dyna, Heliospectra, Sweden) for 6–10 weeks after the emergence of first real leaves (HL). The temperature was around 20 °C. In all experiments, the day/night light rhythm during growth was 8 h/16 h, and fully expanded leaves were used for measurements.

### Photoinhibition measurements

After 8 weeks of growth, detached leaves, with petioles in water, were illuminated for 0–45 min at the PPFD of 2000 µmol m^−2^ s^−1^ at 22 °C or 4 °C in a growth chamber (FytoScope FS 130, Photon Systems Instruments). To block the repair cycle of PSII, leaf petioles were incubated overnight in lincomycin (0.4 mg/ml) solution, under the low irradiance of ~ 10 µmol m^−2^ s^−1^. Before and after the illumination treatment, leaves were kept for 30 min in the dark at 22 °C, after which the chlorophyll *a* fluorescence parameter *F*_v_/*F*_m_ (variable to maximum fluorescence) was measured by Handy Fluorcam FC 1000-H (Photon System Instruments). Weak measuring flashes (620 nm, 10 µs, ~ 1 µmol m^−2^ s^−1^, 19.63 Hz) were used to measure minimal fluorescence (*F*_0_) and an 800-ms saturating pulse (white light, ~ 2000 µmol m^−2^ s^−1^) was fired on the top of the measuring flashes to measure maximal fluorescence (*F*_m_). After the fluorescence measurements, thylakoid membranes from 3 to 6 leaves were isolated as described by Hakala et al. (2005) and immediately stored at − 80 °C. PSII activity was then measured with a parallel method, by measuring the light-saturated (PPFD ~ 4000 µmol m^−2^ s^−1^ from a slide projector) rate of oxygen evolution from the thylakoid membranes (10 µg chlorophyll/ml) at 22 °C in a buffer (40 mM HEPES–KOH (pH 7.6), 1 M betaine monohydrate, 330 mM sorbitol, 5 mM MgCl_2_, 5 mM NaCl, 1 mM KH_2_PO_4_ and 5 mM NH_4_Cl) with an oxygen electrode (Hansatech, King’s Lynn, UK) using 0.5 mM 2,6-dimethylbenzoquinone as an electron acceptor.

The reduction state of *Q*_A_ during the photoinhibition treatments was assayed with PAM-2000 fluorometer (Walz, Effeltrich, Germany). Detached leaves were dark-acclimated for 30 min at room temperature and *F*_0_ and *F*_m_ were measured. After that, the temperature was set to either 22 °C or 4 °C, leaves were placed on wet paper and light was switched on (PPFD of 2000 µmol m^−2^ s^−1^ from a high pressure xenon lamp; Sciencetech Inc.). *F* (fluorescence intensity recorded just before the pulse) and *F*_m_′ (fluorescence intensity during the pulse) were measured during illumination every 15 min by firing an 800-ms saturating pulse (white light, ~ 4000 µmol m^−2^ s^−1^). The quantum yield of PSII in the light was calculated according to Genty et al. (1989), *F*_0_′ (*F*_0_ in light), required for calculation of qP and qL, according to Oxborough and Baker (1997), qP (photochemical quenching) according to Schreiber et al. (1986) and qL (photochemical quenching assuming connected PSII units) according to Kramer et al. (2004).

### Pigments

Chlorophyll and flavonol contents were measured from intact leaves with a nondestructive handheld device (Cerovic et al. 2012; Dualex Scientific, Force-A, Paris, France) after 7–8 weeks of growth. At least three individual uniform-sized NT and CT plants of each accession were selected, from which three leaves were measured. Chlorophyll concentration of isolated thylakoid membranes was measured according to Porra et al. (1989).

### Gas exchange measurements

Net CO_2_ assimilation rates of individual attached leaves of NT and CT plants of each accession, after 7–8 weeks of growth, were measured with a gas exchange measuring system LI-6400-17 (Li-Cor, Biosciences, Linkoln, NE, USA) using a 6400-15 *Arabidopsis* chamber with the aperture diameter of 1 cm (Li-Cor). CO_2_ concentration in the chamber was set to 385 ppm, air humidity to 60 ± 5%, and temperature to 22 °C. Light-acclimated leaves were illuminated first for 2 min at the PPFD of 100 µmol m^−2^ s^−1^ and then for 45 min at the PPFD of 2000 µmol m^−2^ s^−1^.

### ^1^O_2_ and thermoluminescence measurements

The rate of ^1^O_2_ production by isolated thylakoid membranes (100 µg chlorophyll/ml) in high light (PPFD 4000 µmol m^−2^ s^−1^) at 4 °C or at 20 °C, was estimated by measuring the consumption of oxygen, occurring due to the reaction of ^1^O_2_ with 20 mM l-histidine (Sigma-Aldrich, Saint Louis, MO, USA; Rehman et al. 2013) with the oxygen electrode (Hansatech). Oxygen concentration decreases linearly from 5 to 45 s after switching on the high light, during which time the rate of ^1^O_2_ production was calculated. Consumption of oxygen by the thylakoids without added histidine was subtracted from the final results.

^1^O_2_ production by NT, CT and HL Columbia-0 leaves was measured with Singlet Oxygen Sensor Green (SOSG; Invitrogen™). Leaf disks (diameter 6 mm) were vacuum-infiltrated and subsequently incubated overnight in a solution containing 200 µM SOSG and rinsed before the measurements. Leaf disks were illuminated with red light (> 650 nm; PPFD 2000 µmol m^−2^ s^−1^), to avoid ^1^O_2_ production by SOSG itself (Ragás et al. 2009), at 4 °C or at 22 °C. For ^1^O_2_ assays, illumination with red light was interrupted, and SOSG fluorescence was excited by illuminating the leaf with 500 nm light, obtained through a 10 nm bandpass filter (Corion, Newport Corporation), and fluorescence from 535 to 555 nm was recorded with QE Pro spectrometer (Ocean Insights).

Temperature dependencies of the reactions of ^1^O_2_ with SOSG or histidine were tested by illuminating (red light of PPFD 2000 µmol m^−2^ s^−1^ or white light of PPFD 4000 µmol m^−2^ s^−1^, for SOSG and histidine, respectively) methylene blue solution (absorbance at 665 nm = 0.32 or 0.08, for SOSG and histidine, respectively) with 200 µM SOSG or 20 mM histidine at 4 °C or at 22 °C. SOSG fluorescence and oxygen consumption were recorded as above described.

Thermoluminescence bands were recorded with a luminometer from isolated thylakoid membranes (500 µg chlorophyll/ml) as described earlier (Tyystjärvi et al. 2009) in the presence or absence of 20 µM 3-(3,4-dichlorophenyl)-1,1-dimethylurea (DCMU). The thylakoid membranes for ^1^O_2_ and thermoluminescence measurements were isolated as described above from NT and CT plants taken directly from growth chambers.

### Statistical tests and figures

Significances of differences were tested by calculating Student’s *t* test (two-tailed, unequal variances; for calculations and original data, see Online Resource 1). Whenever lincomycin was used, the rate constant of photoinhibition (*k*_PI_) was calculated by fitting the decrease in *F*_v_/*F*_m_ or in the rate of oxygen evolution, as indicated, to the first order reaction equation in SigmaPlot (Systat Software Inc, San Jose, CA, USA). All figures were prepared in SigmaPlot (Systat Software Inc).

## Results

### Assaying photoinhibition of PSII, in the presence of repair, by chlorophyll *a* fluorescence.

**Fig. 1:**
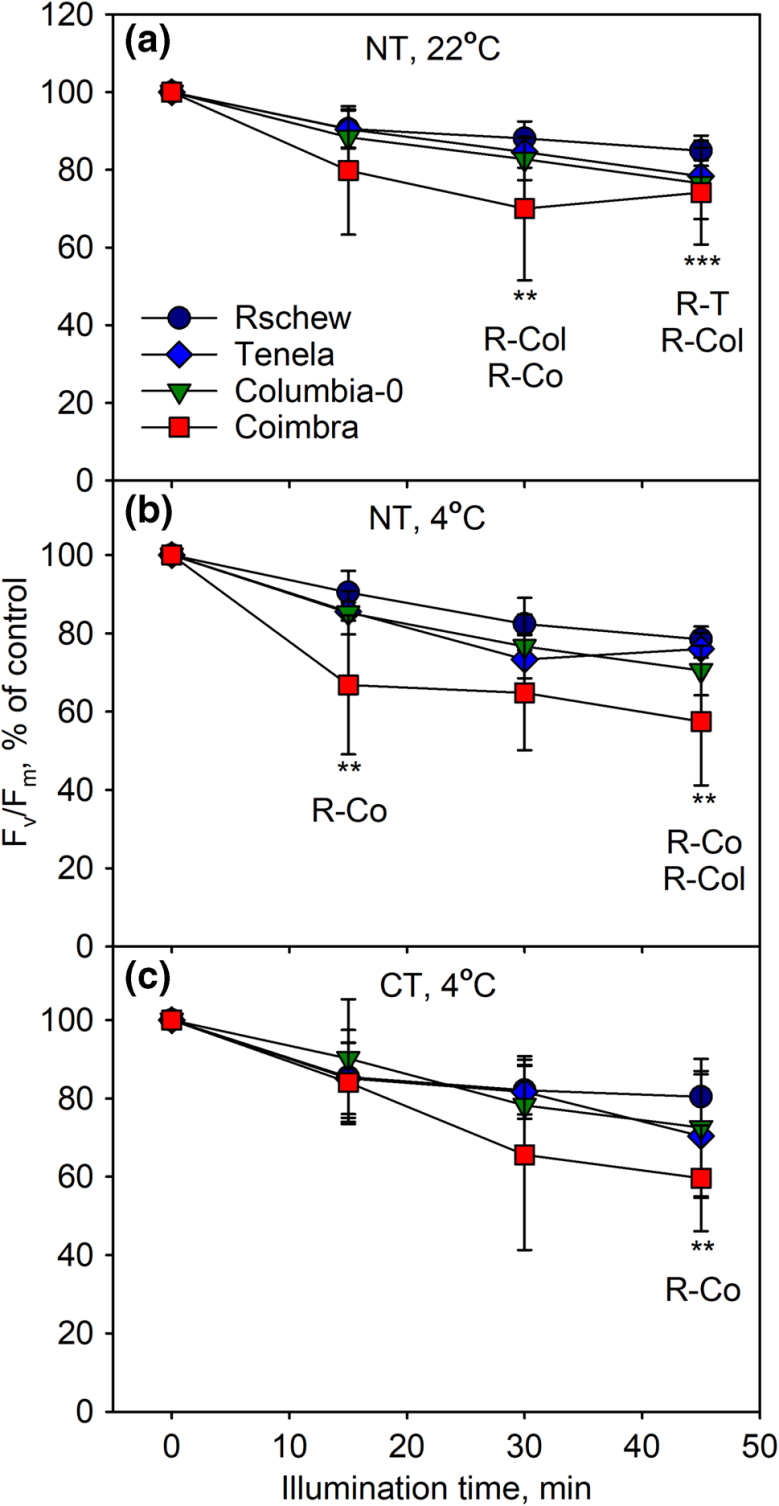
Photoinhibition at 22 °C (**a**) or at 4 °C (**b**, **c**) in the absence of lincomycin, quantified by the chlorophyll *a* fluorescence parameter *F*_v_/*F*_m_. *F*_v_/*F*_m_ was measured from detached leaves of four non-cold-treated (NT; **a**, **b**) or cold-treated (CT; **c**) *A. thaliana* accessions, at different time points during the 45-min illumination (PPFD 2000 µmol m^−2^ s^−1^), after subsequent 30-min dark incubation. The error bars show standard deviations (SD) from at least three biological replicates. Statistically significant differences at any time-point between the indicated accessions are marked with **P < 0.05 or ***P < 0.01. The control values of *F*_v_/*F*_m_ (± SD) were 0.82 (0.03), 0.80 (0.03), 0.82 (0.03) and 0.76 (0.05) for Rschew (R), Tenela (T), Columbia-0 (Col) and Coimbra (Co), respectively, in **a**, 0.82 (0.03), 0.82 (0.03), 0.81 (0.04) and 0.79 (0.06) in **b**, and 0.82 (0.03), 0.79 (0.04), 0.77 (0.08) and 0.79 (0.06) in **c**

Detached leaves from *A. thaliana* accessions (Rschew, Tenela, Columbia-0 and Coimbra) grown at 21 °C for 8 weeks (non-treated; NT) were illuminated with high light (PPFD 2000 µmol m^−2^ s^−1^) either at their growth temperature (at 22 °C) or at 4 °C. Maximum quantum yield of PSII photochemistry (*F*_v_/*F*_m_; after 30 min of dark-incubation) was measured after 0 min, 15 min, 30 min and 45 min in the high light (Fig. 1a, b). Illumination was conducted in the absence of lincomycin to allow the repair of the D1-protein to proceed simultaneously with photoinhibitory damage to PSII. In addition, leaves from plants cold-treated (CT) for 2 weeks at 4 °C were illuminated under the same high light at 4 °C (Fig. 1c). Of the four accessions, Coimbra and Rschew seemed the most susceptible and tolerant to photoinhibition of PSII, respectively (Fig. 1). In NT plants, on the average, *F*_v_/*F*_m_ values declined faster at 4 °C than at 22 °C (Fig. 1a, b). The difference was statistically significant for Tenela after 15 min (*P* = 0.03) and 30 min (*P* = 0.01) and for Rschew after 45 min (*P* = 0.01) of the light treatment. However, the rate of photoinhibition at 4 °C did not differ significantly between NT and CT plants in any of the accessions.

### Assaying photoinhibition of PSII, in the absence of repair, by chlorophyll *a* fluorescence

To determine if the observed differences in photoinhibition were due to differences in the rate of damage to PSII or the rate of repair, we repeated the experiments in the presence of lincomycin. When the repair cycle is blocked with lincomycin, decline in PSII activity can be fitted to the first order reaction equation for calculation of the rate constant of photoinhibition of PSII (*k*_PI_). A good fit was obtained for NT Rschew, Tenela and Columbia-0 at both 22 and 4 °C, but for NT and CT Coimbra and CT Tenela, the fit was of low quality (standard errors of the fits are presented in Online Resource 1).

**Fig. 2:**
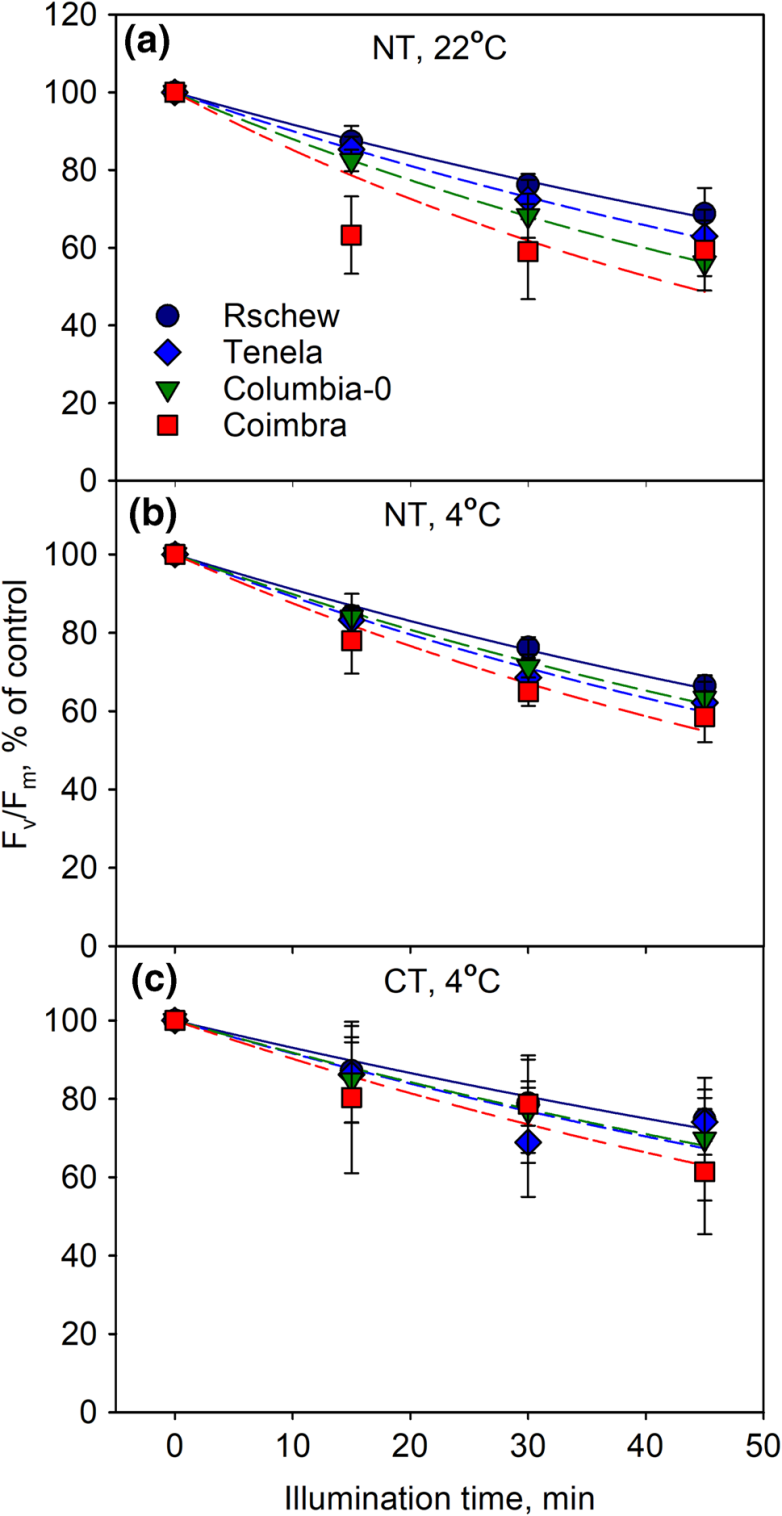
Photoinhibition at 22 °C (**a**) or at 4 °C (**b**, **c**) in the presence of lincomycin, quantified by the chlorophyll *a* fluorescence parameter *F*_v_/*F*_m_. *F*_v_/*F*_m_ was measured from detached leaves of four non-cold-treated (NT; **a**, **b**) or cold-treated (CT; **c**) *A. thaliana* accessions, at different time points during the 45-min illumination (PPFD 2000 µmol m^−2^ s^−1^), after subsequent 30-min dark incubation. The error bars show SD from at least three biological replicates. The lines show a fit to the first order reaction equation. The control values of *F*_v_/*F*_m_ (± SD) were 0.82 (0.03), 0.79 (0.05), 0.83 (0.01) and 0.78 (0.04) for Rschew, Tenela, Columbia-0 and Coimbra, respectively, in **a**, 0.80 (0.01), 0.77 (0.02), 0.81 (0.03) and 0.79 (0.03) in **b** and 0.82 (0.03), 0.77 (0.07), 0.79 (0.06) and 0.79 (0.06) in **c**

Similarly to what was observed without lincomycin (Fig. 1), we found that NT Coimbra and Rschew were, respectively, the least and the most tolerant to photoinhibition, both at 22 °C and at 4 °C (Fig. 2a, b, see Table 1 for the *k*_PI_ values). After the cold-treatment, the differences between accessions were no longer statistically significant (Fig. 2c, Table 1). However, in contrast to the experiments in which the repair was allowed to function, F_v_/F_m_ values declined more rapidly at 22 °C than at 4 °C in 21 °C-grown Columbia-0 (*P* = 0.02) and Coimbra (*P* = 0.05) (Fig. 2a, b). In Rschew and Tenela the differences in *k*_PI_ values were not statistically significant between the temperatures. In all four accessions, photoinhibition at 4 °C proceeded more slowly in CT plants than in NT plants (Fig. 2b, c). Accordingly, the *k*_PI_ values of CT Rschew, Tenela and Coimbra were circa 77% and of CT Columbia-0 84% of those of NT plants (Table 1). The difference was significant in the case of Rschew (*P* = 0.0002) and Tenela (*P* = 0.01).

**Table 1:** The rate constants of photoinhibition (*k*_PI_) in min^−1^ calculated by fitting the decline in *F*_v_/*F*_m_ (Fig. 2) or in PSII-dependent oxygen evolution (Fig. 3), during the 45-min illumination at 22 °C or at 4 °C, to the first order reaction equation in non-cold-treated (NT) and cold-treated (CT) *A. thaliana* accessions

	*k*_PI_, min^−1^ (SD)					
	Non-treated, at 22 °C		Non-treated, at 4 °C		Cold-treated, at 4 °C	
	Fluorescence	Oxygen evolution	Fluorescence	Oxygen evolution	Fluorescence	Oxygen evolution
Rschew	0.0086^a,A^ (0.0011)	0.0194^a,A^ (0.0044)	0.0093^a,A^ (0.0008)	0.0124^a,B^ (0.0044)	0.0072^a,B^ (0.0016)	0.0155^ab,B^ (0.0048)
Tenela	0.0105^b,A^ (0.0012)	0.0210^ab,A^ (0.0024)	0.0115^bc,A^ (0.0013)	0.0192^b,A^ (0.0047)	0.0088^a,B^ (0.0034)	0.0220^a,A^ (0.0048)
Columbia-0	0.0128^c,A^ (0.0014)	0.0247^b,A^ (0.0036)	0.0111^b,B^ (0.0027)	0.0179^ab,B^ (0.0059)	0.0093^a,B^ (0.0042)	0.0128^b,B^ (0.0044)
Coimbra	0.0160^d,A^ (0.0011)	0.0241^ab,A^ (0.0051)	0.0135^c,B^ (0.0027)	0.0226^b,A^ (0.0080)	0.0105^a,B^ (0.0053)	0.0124^b,B^ (0.0044)

SD values (in parentheses) were calculated from at least three biological replicates. Statistically significant differences (*P* < 0.05) between accessions are indicated with lower-case letters and between treatment groups (between NT 22 °C and NT 4 °C, or between NT 4 °C and CT 4 °C) with upper-case letters. Significances of the differences between different accessions are shown only within the same treatment group. Significances of the differences between fluorescence and oxygen evolution data were not calculated

### Assaying photoinhibition of PSII, in the absence of repair, by oxygen evolution

To test whether the results are universal or specific to a particular method of quantification of photoinhibition, we assayed photoinhibition of PSII also by measuring the light-saturated rate of oxygen evolution in the presence of an artificial electron acceptor from thylakoid membranes isolated from the illuminated leaves. Good first-order fits were obtained for NT plants at 22 °C and for NT Rschew, Tenela and Columbia-0 at 4 °C; the fit for NT Coimbra at 4 °C was of low quality, and in all accessions, the first data point measured from CT plants after 15 min of light treatment deviated from first order. The standard errors of the fits are presented in Online Resource 1.

**Fig. 3:**
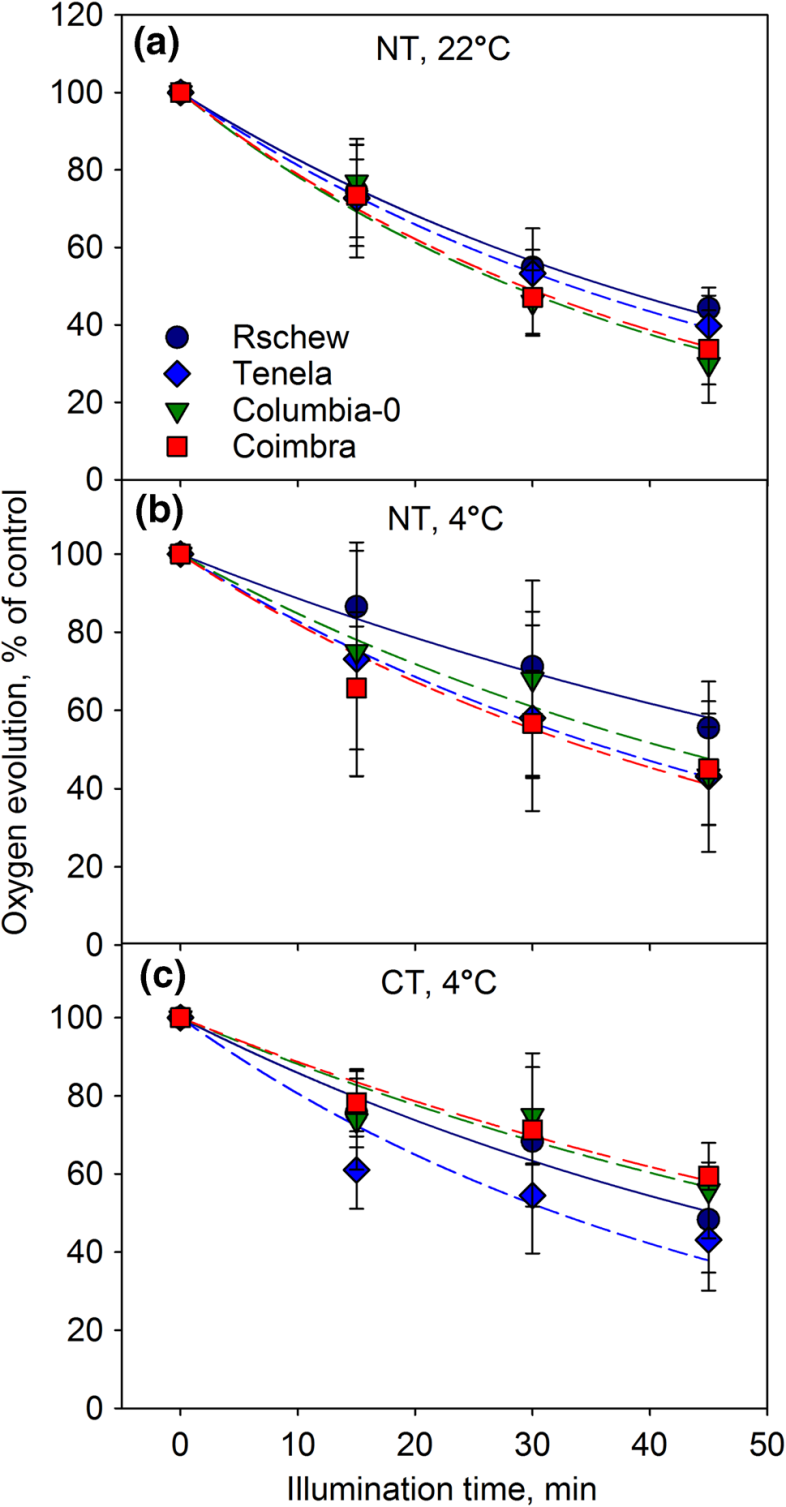
Photoinhibition at 22 °C (**a**) or at 4 °C (**b**, **c**) in the presence of lincomycin, quantified by the light-saturated oxygen evolution of PSII in the presence of an artificial electron acceptor. PSII activity was measured from thylakoid membranes isolated from leaves of four non-cold-treated (NT; **a**, **b**) or cold-treated (CT; **c**) *A. thaliana* accessions, at different time points during the 45-min illumination (PPFD 2000 µmol m^−2^ s^−1^). The error bars show SD from at least four biological replicates. The lines show a fit to the first order reaction equation. The control values in µmol O_2_ (mg chlorophyll)^−1^ h^−1^ (± SD) were 160 (37.5), 162 (26.4), 180 (20.2) and 132 (31.2) for Rschew, Tenela, Columbia-0 and Coimbra, respectively, in **a**, 169 (67.6), 148 (67.1), 162 (37.4) and 135 (24.7) in **b** and 210 (21.7), 186 (22.9), 164 (28.7) and 162 (37) in **c**

Photoinhibition appeared faster when measured with oxygen evolution, in comparison to data obtained with the fluorescence parameter *F*_v_/*F*_m_ (c.f. Figures 2 and 3; Table 1). However, similarly to the fluorescence data, we found that Rschew was the most tolerant accession and Coimbra the least, both at 4 °C and 22 °C (Table 1). Furthermore, photoinhibition proceeded more slowly at 4 °C than at 22 °C in all accessions (Fig. 3a, b), although the differences in the *k*_PI_ values (Table 1) were significant (*P* < 0.05) only for Rschew and Columbia-0. Two-weeks of cold-treatment alleviated photoinhibition at 4 °C statistically significantly only in Coimbra (Fig. 3b, c).

### Physiological parameters of cold-treated and non-treated plants

**Fig. 4:**
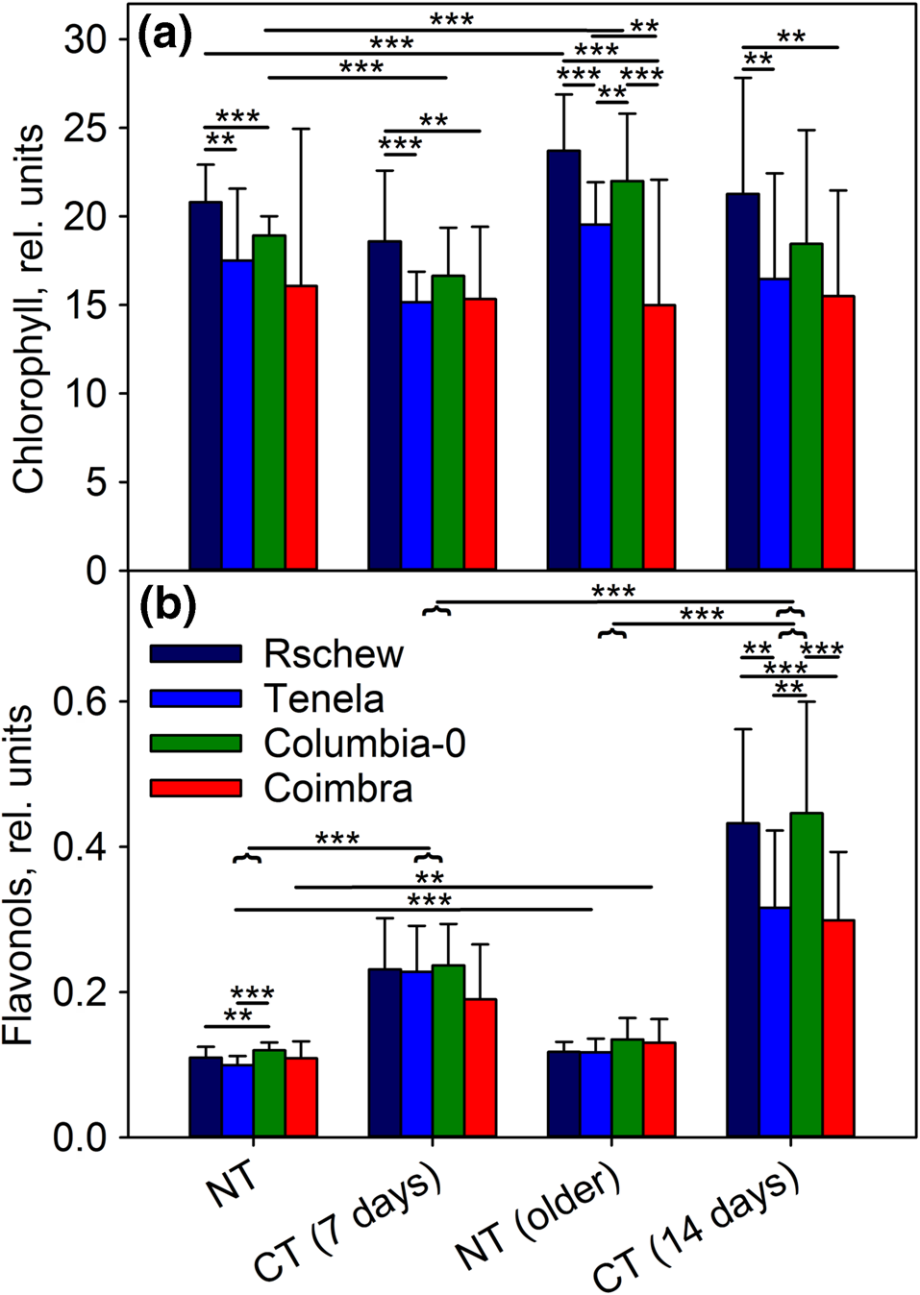
Chlorophyll (**a**) and flavonol (**b**) contents measured from intact leaves of *A. thaliana* accessions (Rschew, Tenela, Columbia-0 and Coimbra) after 7 or 14 days, as indicated, of cold-treatment at 4 °C (CT) or from control plants of similar ages grown at 21 °C (NT). The error bars show SD values from at least four biological replicates. Statistically significant differences are marked with **P < 0.05 or ***P < 0.01 on top of the horizontal lines that show between which samples the difference is significant; the horizontal curly bracket indicates a whole group of four accessions. The significance of the differences between different accessions are shown only within the same treatment group, and significances between NT and CT plants are shown only between the corresponding age groups

To find the factors affecting the different photoinhibition tolerances of the accessions, we measured chlorophyll content, epidermal flavonols and the rate of CO_2_ assimilation non-invasively from intact leaves of NT and CT plants. Chlorophyll contents, measured per leaf area with an optical method, were only little affected by temperature, age or accession. In NT Rschew the chlorophyll content slightly increased during the 14-day time-frame of the experiment (Fig. 4a). The shift of the plants to 4 °C caused a slight decrease in the chlorophyll content after 7 days only in Columbia-0 (Fig. 4a), and even in this accession, no statistically significant difference was anymore observed after 14 days of the cold-treatment. The relative differences in the amounts of chlorophyll between the accessions (Rschew > Columbia-0 > Tenela > Coimbra) stayed the same in all the treatment groups (Fig. 4a).

Contrary to chlorophylls, epidermal flavonols increased significantly during the cold-treatment, both after 7 and 14 days, in all four accessions (Fig. 4b). In Tenela and Coimbra the cold-induced increase in epidermal flavonols was smaller than in Rschew and Columbia-0, and the difference was more obvious after 14 days of cold-treatment (Fig. 4b). In Tenela and Coimbra, flavonols also increased at 21 °C when the NT plants grew older (Fig. 4b).

**Fig. 5:**
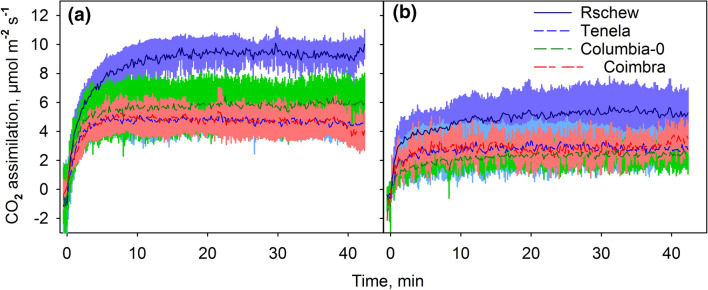
Net CO_2_ assimilation rates at 22 °C during 42-min illumination in high light (PPFD 2000 µmol m^−2^ s^−1^), measured from intact light-acclimated leaves of non-cold-treated (**a**) or cold-treated (**b**) *A. thaliana* accessions (Rschew, Tenela, Columbia-0 and Coimbra). The plants were illuminated with a light intensity close to that of growth light (PPFD 100 µmol m^−2^ s^−1^) for 1 min after which the high light was switched on (at the time point 0). The lines show averages and the colored areas show SD values from at least three biological replicates

To probe the effect of the high-light-illumination on the whole photosynthetic electron transport chain (up to carbon fixation), the rate of net CO_2_ assimilation, during illumination, was measured. Even though PSII activity declined by about 20% during the 45-min high light illumination at 22 °C in the absence of lincomycin (Fig. 1a), a similar illumination treatment did not cause a decline in the rate of net CO_2_ assimilation, measured per leaf area (Fig. 5). Maximum assimilation rates varied between the accessions (Rschew > Columbia-0 > Tenela ≈ Coimbra), and the values of net CO_2_ assimilation (Fig. 5) and the chlorophyll content of the leaves (Fig. 4a) showed similar order between the accessions, especially in NT plants. A 2-week cold-treatment caused a decrease in the maximum rate of CO_2_ assimilation in high light at 22 °C in all four accessions (Fig. 5). The maximum CO_2_ assimilation rate after switching on the high light, however, was reached faster in the CT plants (Fig. 5).

### ^1^O_2_ production in thylakoid membranes and leaves

**Fig. 6:**
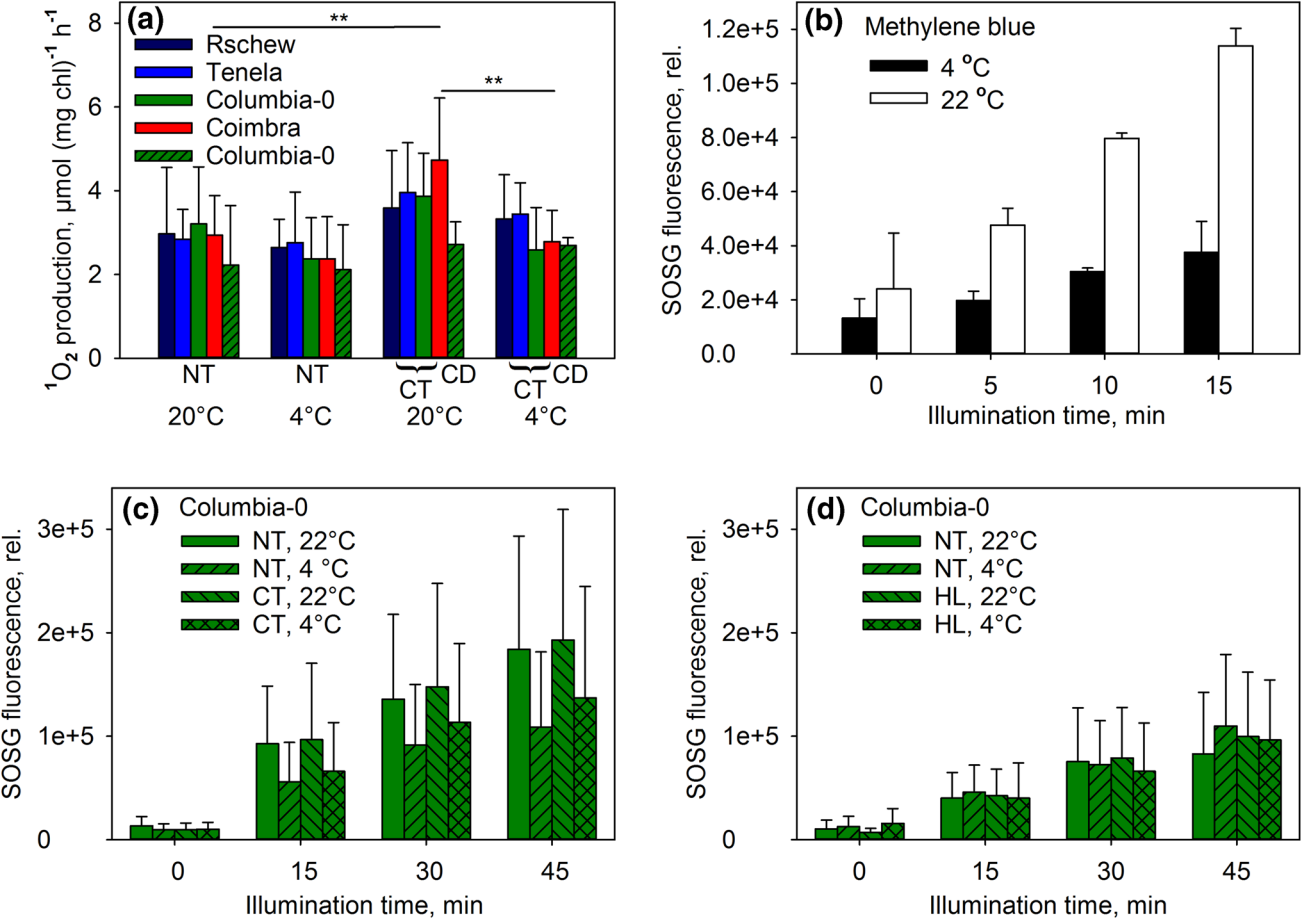
Production of ^1^O_2_ by isolated thylakoid membranes in vitro (**a**), by a methylene blue solution (**b**) and by leaf disks in vivo (**c**, **d**). The measurements were done at 20–22 °C or 4 °C, as indicated, from *A. thaliana* accessions (Rschew, Tenela, Columbia-0 and Coimbra) grown at 21 °C (NT), cold-treated for 2 weeks (CT), grown and developed at 4 °C (CD) or grown in high light at 20 °C (HL). Production of ^1^O_2_ in vitro (**a**) was measured with a histidine-based method in high light (PPFD 4000 µmol m^−2^ s^−1^). ^1^O_2_ production by methylene blue and by *A. thaliana* leaves in vivo (**b–d**) was measured as an increase in SOSG fluorescence after 0–45 min illumination with red light of PPFD 2000 µmol m^−2^ s^−1^ (**b**, **c**) or 1000 µmol m^−2^ s^−1^ (**d**). After each illumination period, SOSG fluorescence was excited with 500 nm light and recorded at 535–555 nm. The error bars show SD values from at least three biological replicates. Statistically significant differences in **a** are marked with **P < 0.05. The significances of the differences between different accessions are shown only within the same treatment group, and significances between NT and CT plants are shown only between the corresponding temperatures

To investigate if the physiological differences of the accessions led to differences in the ^1^O_2_ production capacity of PSII, we measured the rate of ^1^O_2_ production by isolated thylakoid membranes of NT and CT plants, at 20 °C and at 4 °C in high light (Fig. 6a). Undamaged thylakoids were obtained by isolating them directly from growth conditions (21 °C or 4 °C). ^1^O_2_ production was measured with a histidine-based method; the method measures short-term (1–2 min) ^1^O_2_ production upon switching light on. Thylakoids isolated from CT plants produced more ^1^O_2_ at 20 °C than those isolated from NT plants, but the difference was statistically significant only in Coimbra (Fig. 6a). In both NT and CT thylakoids, production of ^1^O_2_ was slightly slower at 4 °C than at 20 °C, but a statistically significant difference was observed only in CT Coimbra. The reaction of histidine with ^1^O_2_, probed by producing ^1^O_2_ with methylene blue, proceeded slightly faster at 20 °C than at 4 °C (Fig. S1), indicating that ^1^O_2_ production by methylene blue is weakly if at all dependent on temperature in the physiological range. Interestingly, the production of ^1^O_2_ by CT thylakoids at 4 °C did not significantly differ from those of NT thylakoids (Fig. 6a). Even thylakoids of Columbia-0 leaves that had grown and developed at 4 °C (CD) did not produce less ^1^O_2_ than thylakoids of NT or CT Columbia-0 (Fig. 6a).

To see if ^1^O_2_ production in vivo would differ from the observed in vitro results, ^1^O_2_ production in high light (PPFD 2000 µmol m^−2^ s^−1^ of red light) was measured from leaf disks of Columbia-0 with SOSG (Fig. 6b–d). The reaction of SOSG with ^1^O_2_, again probed by methylene blue, proceeded over two times as fast at 22 °C than at 4 °C (Fig. 6b). During the illumination of leaves, SOSG fluorescence increased more rapidly at 22 °C than at 4 °C (Fig. 6c). The 2-week cold-treatment did not seem to affect ^1^O_2_ production, as SOSG fluorescence increased similarly in NT and CT leaves (Fig. 6c). To test the effect of growth light intensity, ^1^O_2_ production at 4 and 22 °C was measured also from high light (PPFD 1000 µmol m^−2^ s^−1^) grown (HL) Columbia-0 (Fig. 6d) at PPFD 1000 µmol m^−2^ s^−1^. Expectedly, when illuminated with the lower PPFD of 1000 µmol m^−2^ s^−1^, NT plants produced less ^1^O_2_ than at PPFD 2000 µmol m^−2^ s^−1^. However, SOSG fluorescence increased similarly in NT and HL plants (Fig. 6d). At this PPFD, SOSG fluorescence increased at similar rates at 4 °C and at 22 °C (Fig. 6d). Considering the strong temperature dependence of the reaction between SOSG and ^1^O_2_ (Fig. 6b), the similarity of the increase in SOSG fluorescence at the two temperatures (Fig. 6c, d) suggests that the actual rate of ^1^O_2_ production in NT, CT and HL leaves was higher at 4 °C than at 22 °C, especially at PPFD 1000 µmol m^−2^ s^−1^.

**Fig. 7:**
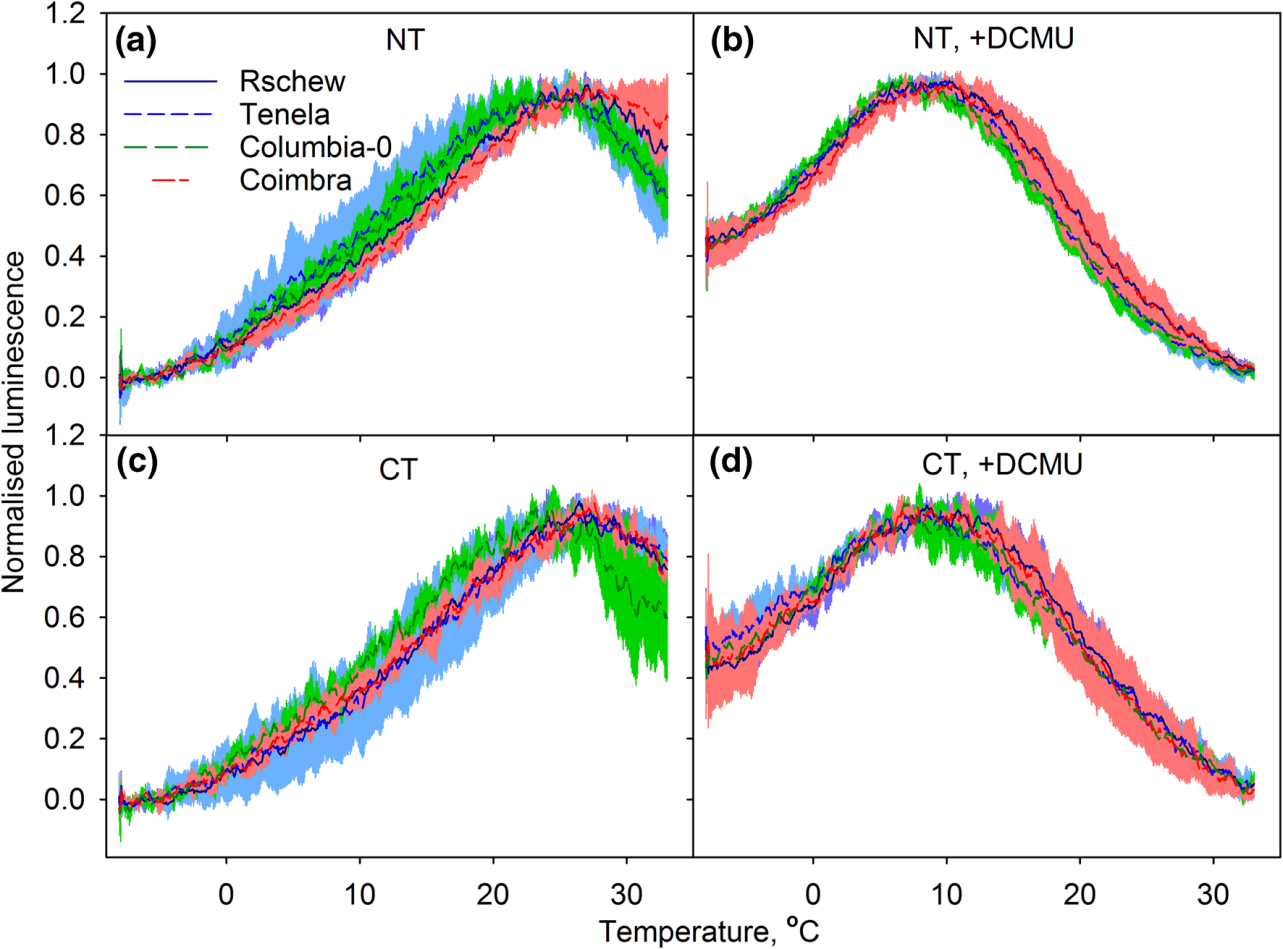
Normalized thermoluminescence bands recorded in the absence (**a**, **c**) or in the presence of DCMU (**b**, **d**) from thylakoid membranes of non-cold-treated (NT; **a**, **b**) or cold-treated (CT; **c**, **d**) *A. thaliana* accessions (Rschew, Tenela, Columbia-0 and Coimbra). The colored areas show SD values from at least three biological replicates. Maximum luminescence intensities (arbitrary units; ± SD) were 2.1 (0.60), 1.8 (0.45), 1.7 (0.15) and 2.8 (0.21) for Rschew, Tenela, Columbia-0 and Coimbra, respectively, in **a**, 2.5 (0.77), 2.2 (0.23), 2.2 (0.17) and 2.6 (0.39) in **b**, 2.0 (0.26), 1.5 (0.20), 1.4 (0.25) and 1.5 (0.2) in **c**, and 1.8 (0.65), 1.2 (0.41), 1.6 (0.16) and 1.9 (0.08) in **d**

Modulation of PSII charge recombination reactions by cold-acclimation (Sane et al. 2003) has been suggested to diminish ^1^O_2_ production (Ivanov 2008). To assess the recombination reactions, thermoluminescence was measured from the same thylakoid membranes used for the ^1^O_2_ assay. In all samples, the peak of the B-band, measured in the absence of DCMU, was between 25 °C and 28 °C whereas the Q-band (measured in the presence of DCMU) peaked around 7–9 °C with no systematic differences between accessions or between NT and CT plants (Fig. 7). However, the thermoluminescence yield of the B-band was 4–46% and the yield of the Q-band 26–46% lower for the CT than NT plants (relative peak intensities are listed in the legend of Fig. 7).

### Excitation pressure at 4 °C and 22 °C

**Fig. 8:**
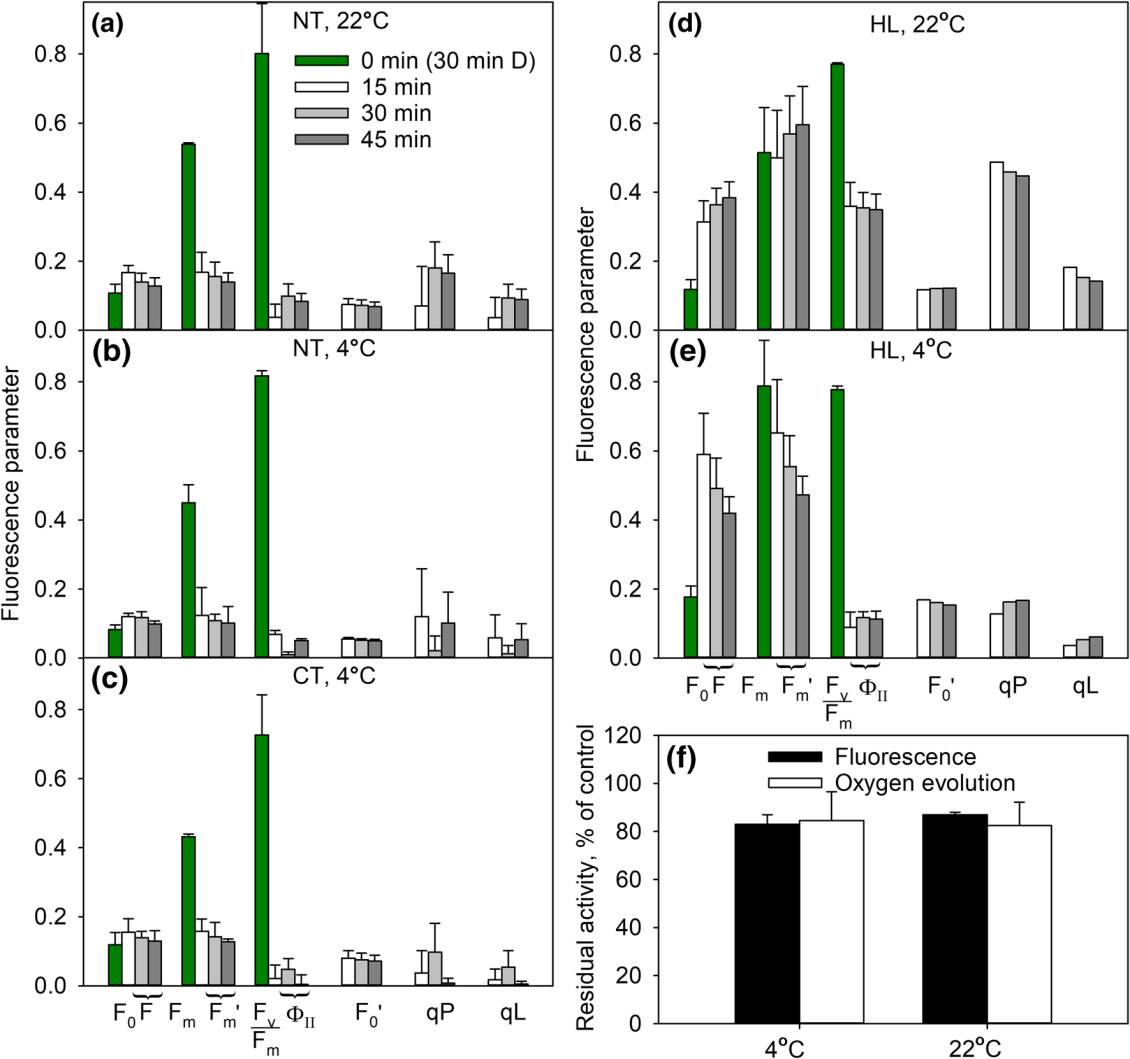
Chlorophyll *a* fluorescence parameters (**a**–**f**) and oxygen evolution (**f**) during 45-min illumination at 22 °C (**a**, **d**, **f**) or at 4 °C (**b**, **c**, **e**, **f**) measured from detached leaves of non-cold-treated (NT; **a**, **b**), cold-treated for 2 weeks (CT; **c**) or grown in high light at 20 °C (HL; **d**, **e**) *A. thaliana* accession Columbia-0. PPFD was 2000 µmol m^−2^ s^−1^ in **a–c** and 1000 µmol m^−2^ s^−1^ in **d**–**f**. *F*_0_ and *F*_m_ were measured after 30 min dark-acclimation [time point 0 min (30 min D)] and all other parameters were measured in the light, 15, 30 or 45 min after switching on the light, as indicated (**a**–**e**). *F* is the fluorescence yield under illumination. *F*_v_/*F*_m_ is the maximum quantum yield of PSII electron transfer, calculated as (*F*_m_ − *F*_0_)/*F*_m_. ɸ_II_ is the quantum yield of PSII electron transfer in the light, calculated as (*F*_m_' − *F*)/*F*_m_'. *F*_0_′ is the value of F_0_ in the light, calculated as 1/(1/*F*_0_ − 1/*F*_m_ + 1/*F*_m_'). qP and qL are estimates of photochemical quenching; qP is calculated as (*F*_m_' − *F*)/(*F*_m_' − *F*_0_′) and qL as qP × *F*_0_′/*F*. *F*_0_, *F*_m_ and *F*_m_' values may differ between treatment groups due to different settings of the fluorometer. Occasional below-zero qP or qL values in (**a**–**c**) were interpreted as zeros. HL Columbia-0 leaves were incubated overnight with the petioles in lincomycin (**d**–**f**), and photoinhibition after the 45-min illumination was quantified by the chlorophyll *a* fluorescence parameter *F*_v_/*F*_m_ from leaves after 30 min in dark and by the light-saturated oxygen evolution of PSII in the presence of an artificial electron acceptor measured from thylakoids isolated from the illuminated leaves (**f**). The error bars show SD from at least three biological replicates. In **d**, **e** *F*_0_′, qP and qL have been calculated from averages

To understand the role of excitation pressure in photoinhibition, the reduction state of *Q*_A_ during 45-min high-light treatment was measured from Columbia-0 (Fig. 8). In NT plants illuminated at PPFD 2000 µmol m^−2^ s^−1^, the yield of PSII electron transfer and coefficients of photochemical quenching, qP and qL, were slightly lower at 4 °C than at 22 °C, and the difference was statistically significant (*P* < 0.05) at the 30-min time point (Fig. 8a, b). The data indicate that *Q*_A_ was almost completely reduced at 4 °C in both CT and NT Columbia-0 (Fig. 8b, c). To obtain plants with a larger difference in the excitation pressure between the two temperatures, we repeated the experiments under PPFD 1000 µmol m^−2^ s^−1^ with HL Columbia-0 (grown at PPFD 1000 µmol m^−2^ s^−1^) in the presence of lincomycin. In this case, a much higher proportion of *Q*_A_ remained reduced at 4 °C than at 22 °C (Fig. 8d, e). To explore the relationship between excitation pressure and photoinhibition of PSII, light-induced damage to PSII was quantified from the HL plants after 45-min illumination at 1000 µmol m^−2^ s^−1^ in the presence of lincomycin, using both fluorescence and oxygen evolution methods. The oxygen evolution capacity of PSII declined slightly faster at 22 °C than at 4 °C, but the difference was not statistically significant (Fig. 8f). *F*_v_/*F*_m_ values, in turn, indicated slightly faster photoinhibition at 4 °C (*P* = 0.046; Fig. 8f).

## Discussion

### Rate of photodamage in vivo is slower or equal at 4 °C than at 22 °C, in plants grown at 21 °C

Temperature affects the rate of the damaging reaction of photoinhibition. We used two parallel methods for measuring photoinhibition of PSII: the chlorophyll *a* fluorescence parameter, *F*_v_/*F*_m_, and PSII-dependent oxygen evolution measured from thylakoids isolated from illuminated leaves. Comparison of the present data obtained from non-treated plants grown at 21 °C (NT) of all accessions showed a trend of slower damage to PSII in the presence of lincomycin at 4 °C than at 22 °C (Table 1). Fluorescence data showed a larger *k*_PI_ at 22 °C than 4 °C for Columbia-0 and Coimbra and oxygen evolution data for Rschew and Columbia-0 (Table 1; Fig. 2). However, the experimental noise in both *F*_v_/*F*_m_ and oxygen data does not allow more than identification of a trend. In no case was the *k*_PI_ value significantly larger at 4 °C than 22 °C (Table 1). A slight increase in the rate of the damaging reaction at higher temperatures corroborates earlier in vitro (Tyystjärvi et al. 1994) and in vivo (Tyystjärvi 2013) results. Values of PSII electron transfer yield and photochemical quenching, measured in Columbia-0, indicate that *Q*_A_ was more reduced at 4 °C than at 22 °C during the 45-min high light treatment (Fig. 8a, b). Thus, the finding that the damaging reaction of photoinhibition is faster at the higher, not at the lower temperature, indicates that the excitation pressure hypothesis, according to which the damage to PSII is caused by reduction of the electron transfer chain (see Kornyeyev et al. 2003), does not explain the temperature dependence of photoinhibition of PSII in our data.

The use of a high PPFD for photoinhibition treatment, as done in our study, is justified by the finding that *k*_PI_ is directly proportional to PPFD (for a review, see Tyystjärvi 2013). Therefore, when comparing different samples, the choice of treatment PPFD is somewhat irrelevant. However, due to high intensity of light used in the treatments, the difference in the reduction state of *Q*_A_ between 4 and 22 °C was small in NT plants, possible because they were grown under much lower light intensity. Therefore, we quantified both excitation pressure and photoinhibition also from high-light-grown (HL) Columbia-0 accession. The 45-min high light treatments were given at the growth light of the HL plants, both at the growth temperature (22 °C) and at 4 °C. In the case of HL plants, *Q*_A_ was clearly more reduced in high light at 4 °C than at 22 °C (Fig. 8d, e). Despite the big difference in excitation pressure, photoinhibition rate, measured as a decline in the oxygen evolution capacity of PSII, did not differ much between the two temperatures (Fig. 8f). However, *F*_v_/*F*_m_ measurements showed faster photoinhibition at lower temperature, even though the difference was small (Fig. 8f).

Tsonev and Hikosaka (2003) and Kornyeyev et al. (2003) observed significantly faster decline in *F*_v_/*F*_m_ at low temperature than at optimal temperature in vivo in *Chenopodium album* and *Gossypium hirsutum*, respectively, at several PPFD values. It is possible that the discrepancy between our results and this negative temperature dependence originates from the methods used for assessing photoinhibition. *F*_v_/*F*_m_ may reflect low-temperature-induced fluorescence quenching that does not relax during a typical dark-adaptation time (20‒60 min). Such slowly relaxing decline of *F*_v_/*F*_m_ might be related to the sustained NPQ that develops in some evergreen species at low temperatures (for a review, see Verhoeven 2014) and might be important also in *A. thaliana* (Malnoë et al. 2018). Even in our data, *F*_v_/*F*_m_ results from HL plants alone (Fig. 8f) might allow concluding that excitation pressure enhances photoinhibition. Due to these complications, the use of methods based on oxygen evolution of PSII as a measure of photoinhibition is advisable, especially in studies involving cold-treatments.

Upon a (sudden) decrease in temperature, light absorption continues but carbon fixation and other enzymatic reactions slow down. Therefore, exposure to high light at low temperature can induce severe stress for plants (see e.g. Alboresi et al. 2011), even though the rate of light-induced damage to PSII would not be accelerated compared to optimal conditions. In non-acclimated plants, low temperature commonly slows down the enzymatic reactions of concurrent recovery of photoinhibitory damage (Greer et al. 1986). In addition, ROS produced at low temperatures may specifically inhibit translation in the chloroplast (Kojima et al. 2009). Therefore, net loss of PSII activity may occur (Öquist et al. 1993; Allakhverdiev and Murata 2004). The results of the present study confirm faster loss of PSII activity at 4 °C than at 22 °C in NT plants when no protein synthesis inhibitor was used (Fig. 1a, b). As the rate of the damage to PSII was faster at 22 °C than at 4 °C (Table 1), these results simply confirm that the repair cycle of PSII functioned more slowly at 4 °C than at 22 °C.

### Two-week cold-treatment may increase photoinhibition tolerance in *A. thaliana* accessions

Two-week cold-treatment at 4 °C slowed down photoinhibition of PSII by slowing down the damaging reaction of photoinhibition of PSII in all accessions when measured with *F*_v_/*F*_m_, and the difference was statistically significant in the two most cold-tolerant accessions Rschew and Tenela (Fig. 2; Table 1), similarly as reported previously for cyanobacteria (Vonshak and Novoplansky 2008). Oxygen evolution data, in turn, showed protection by the cold-treatment only in the accessions Columbia-0 and Coimbra, which can be considered as intermediate cold-tolerant and cold-susceptible, respectively. Knaupp et al. (2011) observed that 14 days of similar cold-treatment enhanced stability of PSII during consequent freezing and thawing, and accordingly, control activities of thylakoids isolated from CT plants were higher than those of NT plants (see the legend of Fig. 3). This, however, would not affect the measured in vivo photoinhibition rate because photoinhibition is a first-order reaction (Tyystjärvi and Aro 1996). To conclude, 2-week cold-treatment enhanced photoinhibition-tolerance in some *A. thaliana* accessions, even though the effect was rather small in the present study.

Duration of the cold-treatment has to be taken into account when comparing the present results with literature (Gray et al. 1996; Krause et al. 1999; Savitch et al. 2000; Venema et al. 2000; Sane et al. 2003; Yang et al. 2010). Freezing tolerance is enhanced rapidly upon a cold-treatment (Ristic and Ashworth 1993) but, for example, low-temperature grown *A. thaliana* plants are reported to be able to keep *Q*_A_ more oxidized than plants that have been shifted to the low temperature (Savitch et al. 2001). Accordingly, we did not observe alleviation in *Q*_A_ reduction during the 45-min high light treatment at 4 °C in CT Columbia-0 compared to NT Columbia-0 (Fig. 8c). Also, leaves fully developed at a low temperature were found to be more tolerant against photoinhibition than leaves cold-treated for 20 days (e.g. Gray et al. 2003).

Direct comparison between NT and CT plants in the present study is complicated by the fact that the plants might be at slightly different developmental stages. For example, chlorophyll content (per leaf area) increased in NT plants at 21 °C during the 2-week measurement period while in CT plants there was very little or no increase in chlorophyll during the 2 weeks at 4 °C (Fig. 4). It has been shown that younger leaves are more susceptible to photoinhibition but in fully developed leaves, such as those used in the present study, the differences may be small (Bielczynski et al. 2017). Therefore, the differences in photoinhibition tolerance between accessions in the present study can be assumed to be due to the cold-treatment and not because of the developmental stage of a leaf.

Decrease in the amount of functional PSII units during exposure to the 45-min illumination with strong light at 22 °C (Figs. 1‒3) did not lead to a decrease in net CO_2_ assimilation (Fig. 5). The lack of change in the rate of carbon fixation during the illumination, both in NT and CT plants, suggests that *A. thaliana* leaves contain more PSII than needed to saturate the needs of the carbon fixation reactions in high light (see also Chow 2001). Net CO_2_ assimilation at 22 °C was slower in CT leaves than in their NT counterparts (Fig. 5), similarly as observed previously after a 6-day cold-treatment (Velitchkova et al. 2020). No recovery occurs after 5 days in optimal conditions (Velitchkova et al. 2020), suggesting that short cold-treatments cause cold stress or induce persistent down-regulation of carbon fixation in *A. thaliana*.

### Decrease in thermoluminescence yield did not lower ^1^O_2_ production after 2-week cold-treatment

What causes the improvement of photoinhibition-tolerance by exposure to low temperatures? In plants, ^1^O_2_ is produced mainly in the recombination reactions of PSII, and ^1^O_2_ has been suggested to cause photodamage to PSII (e.g. Vass and Cser 2009). Increased production of ^1^O_2_ in the *npq1lut2* mutant of *A. thaliana* leads to oxidative damage to thylakoid proteins in high light at 10 °C (Alboresi et al. 2011). In different plant species including *A. thaliana* (Janda et al. 2000; Ivanov et al. 2001; Sane et al. 2003), cold-acclimation causes a decrease in the redox gap between the *Q*_A_ and *Q*_B_ electron acceptors of PSII, which favors a direct, non-radiative recombination route (Rappaport and Lavergne 2009) for the elimination of the charge in *Q*_A_^−^ (Ivanov 2008). The direct route does not produce ^1^O_2_ and, therefore, the observed decrease in photoinhibition caused by cold-acclimation might be due to a decrease in ^1^O_2_ production (for a more comprehensive discussion about the relationship between recombination reactions and ^1^O_2_, see Vass and Cser 2009). Accordingly, Ramel et al. (2012) observed a decrease in both production of ^1^O_2_ and oxidation of beta-carotene after 99 h at 7 °C. Unfortunately, ^1^O_2_ was measured with SOSG in white light, a condition that has been shown to induce ^1^O_2_ production by the sensor itself (e.g. Ragás et al. 2009).

We did not observe shifts in the peak temperatures of the thermoluminescence bands (Fig. 7), contrary to the previous observations (Sane et al. 2003). The shifts were observed already after 7 days of cold-treatment (Sane et al. 2003). It might be possible that temperature induced modifications of PSII are observed only when thermoluminescence is measured from leaves, as done by Sane et al. (2003), but not from isolated thylakoids as used in the present study. We did, however, observe a decrease in the thermoluminescence yield for both B and Q-bands after the 2-week treatment at 4 °C, resembling that reported by Ivanov et al. (2001) and Sane et al. (2003). The intensity of the B-band decreased only little, ~ 4%, in Rschew but the decrease was more pronounced in Tenela (18%), Columbia-0 (20%) and Coimbra (46%). We also observed strong alleviation of photoinhibition in CT Coimbra, compared to NT Coimbra. However, in other accessions the decrease in the thermoluminescence peak intensity did not correlate with the rate of photoinhibition (Fig. 7, Table 1). Furthermore, cold-treatments did not cause a decrease in the intrinsic capacity of the thylakoids (presumably PSII) to produce ^1^O_2_ during illumination (Fig. 6). Neither was ^1^O_2_ production in vivo in Columbia-0 affected by 2-week cold-treatment or by growth under high light (PPFD 1000 µmol m^−2^ s^−1^) at 20 °C. To conclude, the present cold-treatment at rather low light, or growth at high light but at optimal temperature, did not lead to diminished ^1^O_2_ production. Possibly a combination of high light and low temperature is needed to alter the redox properties of PSII in *A. thaliana* (see also Velitchkova et al. 2020). We cannot, however, exclude the possibility that the in vivo results are affected e.g. by different diffusion of SOSG into NT, CT or HL leaves, due to the different optical and structural properties of the leaves.

If the rate of repair is insignificant at low temperatures, then results from illumination treatments without an inhibitor of (chloroplast) protein synthesis could be taken to represent the amount of damage to PSII. However, in the present data, addition of lincomycin clearly increased photoinhibition, indicating that the repair cycle is active at 4 °C even in NT plants (Figs. 1, 2). Also the finding that genes coding for a protease involved in PSII repair cycle (FtsH) are up-regulated at a low temperature (Soitamo et al. 2008) supports this view. Consequently, it remains unclear whether diminished ^1^O_2_ production by modified recombination reactions (Ivanov et al. 2001; Sane et al. 2003) decreases PSII damage or protects the repair reactions (see e.g. Kojima et al. 2009).

### Sensitivity to ROS may not equal sensitivity to photoinhibition of PSII

The roles of ROS in the low-temperature-induced protection against photoinhibition are interesting also for the mechanism of photoinhibition. The *k*_PI_ values of the four accessions, measured by both *F*_v_/*F*_m_ and oxygen evolution, were mostly in the order Rschew < Tenela < Columbia-0 < Coimbra (Table 1). Furthermore, Coimbra was more susceptible to the damage caused by high light than the other accessions when the PSII repair cycle was allowed to run (Fig. 1). Thus, the two cold-tolerant accessions (Rschew and Tenela) were somewhat more tolerant to photoinhibition than the other two accessions (Columbia-0 and Coimbra). Previously, it has been shown that Tenela is sensitive to oxidative stress (Brosché et al. 2010), which is probably linked to increased production of hydrogen peroxide at low temperatures (Distelbarth et al. 2013). However, non-acclimated Tenela was not particularly sensitive to photoinhibition of PSII (Figs. 1, 2, 3), which supports the suggestion that oxidative stress and photoinhibition are not (always) related (Hakkila et al. 2014). In fact, the rate of light-induced damage to PSII may not be defined by ROS but rather by direct light absorption of the oxygen-evolving complex of PSII (Hakala et al. 2005).

In vivo, ^1^O_2_ production may be affected e.g. by the reduction state of *Q*_A_. We measured in vivo ^1^O_2_ production with SOSG, from leaves of Columbia-0. At PPFD 1000 µmol m^−2^ s^−1^, the rate of ^1^O_2_ production in high light seemed faster at 4 °C than at 22 °C (considering the strong temperature dependence of the reaction between SOSG and ^1^O_2_; Fig. 6b), coinciding with a big difference in the reduction state of *Q*_A_ between the temperatures (Figs. 6 and 8). At PPFD 2000 µmol m^−2^ s^−1^, when the difference in *Q*_A_ reduction was small, also the difference in ^1^O_2_ production between the temperatures was smaller. The results support the idea that the reduction of *Q*_A_ enhances ^1^O_2_ production. However, photoinhibition proceeded rather similarly or faster at 22 °C compared to 4 °C (Figs. 2, 3, 8f).

The sensitivity to oxidative stress may also be governed by a genetic program (Brosché et al. 2010). Hydrogen peroxide, which has a relatively long lifetime in cells, is an important signaling molecule (for a review, see e.g. Černý et al. 2018). Further complications in the roles of ROS in cold-acclimation are exemplified by discrepant findings on the relationship between ROS metabolism and freezing tolerance (Distelbarth et al. 2013; Hashempour et al. 2014).

### Do flavonols quench ^1^O_2_ and/or protect plants from photoinhibition?

Synthesis of many flavonol species is induced in coldness (e.g. Schulz et al. 2015), and their amount was shown to correlate with freezing tolerance (Korn et al. 2008). Flavonols are also able to physically and chemically quench ^1^O_2_ in vitro (Tournaire et al. 1993), and it has been suggested that they function as ^1^O_2_ quenchers also in vivo (Majer et al. 2014). Accordingly, Havaux and Kloppstech (2001) reported that mutants unable to synthetize anthocyanins and flavonols had lower *F*_v_/*F*_m_ values and more lipid peroxidation at low temperatures than wild-type plants.

**Fig. 9:**
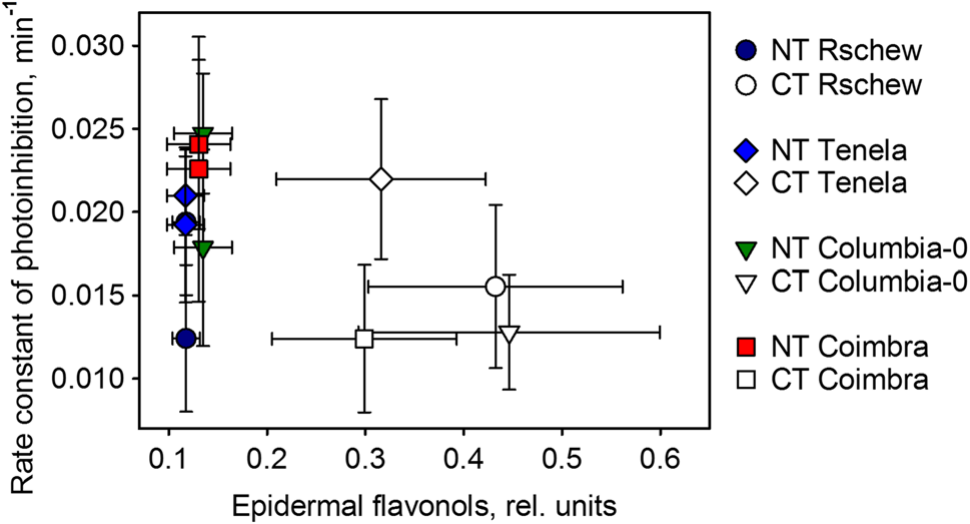
Rate constants of photoinhibition (quantified as a loss of oxygen evolution capacity of PSII) plotted against the amount of epidermal flavonols of four *A. thaliana* accessions (Rschew, Tenela, Columbia-0 and Coimbra). Values are measured after 2 weeks of cold-treatment at 4 °C (CT) or from control plants of similar ages grown at 21 °C (NT). The data are from Fig. 4 and from Table 1

In Columbia-0 and Coimbra one can see a negative correlation between flavonol amounts and the rate constant of photoinhibition, suggesting that flavonols can in some accessions protect against photoinhibition (Fig. 9). However, no general correlation was found. In Columbia-0, cold-acclimation did not decrease ^1^O_2_ production in vivo (Fig. 6). Of course, the optical method used in the present study reflects mostly epidermal flavonols and, therefore, the possible effects of chloroplast-located flavonols (probably in small amounts compared to epidermal flavonols) able to quench ^1^O_2_ (Agati et al. 2007) may not be observed. In addition, it has been shown that some flavonol species are more efficient in quenching ROS than others (Majer et al. 2014). Therefore, a method measuring the total amount of flavonols, as used here, may not give a full answer. To resolve the roles of flavonols in cold-tolerance, more research is required.

#### *Author contribution statement*

ET, KBM and HM conceived and designed research. HM, KBM, IK, AM, KN and DŠ conducted experiments. HM, ET and KBM analyzed data. HM wrote the manuscript with contributions from ET and KBM. All authors read and approved the manuscript.

## Electronic supplementary material

Below is the link to the electronic supplementary material.

Fig. S1 Consumption of oxygen in high light (PPFD 4000 µmol m-2 s-1) at 20 °C or 4 °C, reflecting 1O2 production by a methylene blue solution (calculated per optical density (OD) at 665 nm = 1), in the presence and absence of histidine (TIF 94 kb)

Supplementary file2 (XLSX 1844 kb)

## Abbreviations

CD: Developed at cold (grown 15 weeks at 4 °C)
CT: Cold-treated (grown 6 weeks at 21 °C and 2 weeks at 4 °C)
DCMU: 3-(3,4-Dichlorophenyl)-1,1-dimethylurea
*F*_v_/*F*_m_: Ratio of variable to maximum fluorescence
HL: High light (grown 6–10 weeks under PPFD of 1000 µmol m^−^^2^ s^−^^1^ at 21 °C)
*k*_PI_: Rate constant of photoinhibition of PSII
NT: Non-treated (grown at 21 °C)
NPQ: Non-photochemical quenching (of fluorescence)
ROS: Reactive oxygen species
^1^O_2_: Singlet oxygen
SOSG: Singlet Oxygen Sensor Green
